# The dynamics of linear polyubiquitin

**DOI:** 10.1126/sciadv.abc3786

**Published:** 2020-10-14

**Authors:** Alexander Jussupow, Ana C. Messias, Ralf Stehle, Arie Geerlof, Sara M. Ø. Solbak, Cristina Paissoni, Anders Bach, Michael Sattler, Carlo Camilloni

**Affiliations:** 1 Department of Chemistry and Institute for Advanced Study, Technical University of Munich, Garching 85747, Germany.; 2 Institute of Structural Biology, Helmholtz Zentrum München, Neuherberg 85764, Germany.; 3 Center for Integrated Protein Science Munich at Department of Chemistry, Technical University of Munich, Garching 85747, Germany.; 4 Department of Drug Design and Pharmacology, Faculty of Health and Medical Sciences, University of Copenhagen, Universitetsparken 2, DK-2100 Copenhagen, Denmark.; 5 Dipartimento di Bioscienze, Università degli studi di Milano, 20133 Milano, Italy.

## Abstract

Polyubiquitin chains are flexible multidomain proteins, whose conformational dynamics enable them to regulate multiple biological pathways. Their dynamic is determined by the linkage between ubiquitins and by the number of ubiquitin units. Characterizing polyubiquitin behavior as a function of their length is hampered because of increasing system size and conformational variability. Here, we introduce a new approach to efficiently integrating small-angle x-ray scattering with simulations allowing us to accurately characterize the dynamics of linear di-, tri-, and tetraubiquitin in the free state as well as of diubiquitin in complex with NEMO, a central regulator in the NF-κB pathway. Our results show that the behavior of the diubiquitin subunits is independent of the presence of additional ubiquitin modules and that the dynamics of polyubiquitins with different lengths follow a simple model. Together with experimental data from multiple biophysical techniques, we then rationalize the 2:1 NEMO:polyubiquitin binding.

## Teaser

A new efficient method for SAXS-driven simulations allows researchers to explain the dynamics of linear polyubiquitin.

## INTRODUCTION

Ubiquitination is a reversible posttranscriptional modification system that regulates key physiological processes, such as protein degradation, cell cycle, apoptosis, DNA repair, and signal transduction (*1*–*3*). Once a protein substrate is monoubiquitinated (e.g., a lysine of the substrate is conjugated through an isopeptide bond to the C terminus of a ubiquitin monomer), an additional ubiquitin may be conjugated to either one of the seven lysine residues of the first ubiquitin (K6, K11, K27, K29, K33, K48, and K63) (*4*) or its N-terminal methionine residue (M1) (*5*–*7*). This process can lead to the assembly of polyubiquitin chains of various lengths and topologies. The resulting polymeric chains are then associated with different cellular mechanisms (*8*). Since all these polymers are made of the same single unit, the highly conserved 76-residue-long ubiquitin domain, the ubiquitin code is an example of a conformation-based alphabet, where both the polymerization site (*8*, *9*) and the chain length (*10*) regulate the recognition by different partners and thereby determine the cellular fate of the protein. The role of polyubiquitin length and dynamics in molecular recognition processes is poorly understood (*8*, *10*, *11*). An overall assessment of the typical length of different polyubiquitin chains in physiological conditions is missing, and only sporadic indications are available. For example, in the case of K48-linked polyubiquitin, a length of four is generally considered optimal for molecular recognition of the 26*S* proteasome (*12*), while the nuclear protein localization protein 4 is selective for K48-linked chains longer than six (*13*). It was reported that K48-linked tetraubiquitin (Ub_4_) slows down further ubiquitination (*14*–*16*), while this is not the case for K63-linked Ub_4_ (*16*).

**Fig. 1:**
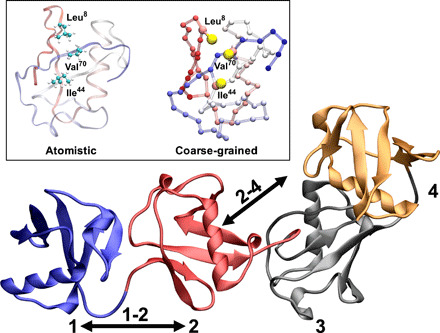
Schematic illustrations linear polyubiquitins. Cartoon representation of linear tetraubiquitin; the ubiquitin domains are numbered from the N terminus to the C terminus from 1 to 4. The inset shows an atomistic and coarse-grained (CG) (Martini) representation of the hydrophobic patch (Ile^44^, Val^70^, and Leu^8^) of the ubiquitin domain.

Linear M1-linked polyubiquitin chains (Fig. 1), whose assembly is catalyzed by linear ubiquitin chain assembly complex (LUBAC) (*5*), have been shown to play a role in inflammation, immune responses, and oncogenesis (*17*–*19*). Their most studied function is the involvement in the activation of the canonical nuclear factor κB (NF-κB) pathway (*6*, *7*, *17*, *20*–*23*). In this pathway, the IKK complex [or IκB (inhibitor of the NF-κB proteins) kinase, formed by IKKα, IKKβ, and NEMO, also known as IKKγ, the NF-κB essential modulator] is activated by LUBAC upon activation by various stimuli (*22*). LUBAC preferentially recognizes and conjugates linear ubiquitin chains on NEMO. NEMO also has a specific linear diubiquitin-binding region referred to as the “ubiquitin binding in A20-binding inhibitor of NF-kappa-B activation (ABIN) and NEMO” (UBAN) motif (*24*), which forms a helical coiled-coil dimer in solution (*23*). Recognition of a linear polyubiquitin conjugated to NEMO by the UBAN domain of another NEMO may trigger the clustering of the IKK complex and conformational changes that subsequently activate IKK (*25*, *26*). Once active, IKK can phosphorylate and inactivate the IκBs, leading to the release of NF-κB (*27*). It was recently shown that it is possible to inhibit NF-κB activation upon UBAN-dependent tumor necrosis factor–α and T cell receptor/CD28 stimulation by small molecules that inhibit the binding of linear polyubiquitins to the NEMO_UBAN_ domain (*23*). While the NEMO_UBAN_ domain can bind linear diubiquitin, it has been observed that full-length NEMO can only bind Ub_4_ or longer, suggesting a length-dependent activation mechanism (*21*). Furthermore, another study suggested that the binding of NEMO to chains of 10 linear ubiquitins or longer induces a different conformation of NEMO compared to the binding of shorter chains (*20*).

Characterizing the conformational space of polyubiquitin chains as a function of length is critical to understand their physiological behavior. Such structural characterization is nonetheless very challenging. Polyubiquitins, from diubiquitin to longer chains, exhibit a very dynamic behavior (*28*) that requires determining a statistical ensemble of all the relevant configurations populated in solution. The combination of molecular dynamics (MD) with experimental small-angle x-ray scattering (SAXS) data is very well suitable to study dynamic protein systems (*29*) including polyubiquitin of varying chain size. SAXS does not provide high-resolution structural information. Conversely, MD simulations may be used to determine the statistical ensemble of configuration populated by a system in equilibrium condition, but a full modeling base on MD simulations is hampered by the size of the system (*30*, *31*). This problem can, in principle, be alleviated by coarse-grain force fields (*32*), eventually combined with enhanced sampling techniques (*33*), that can massively speed up MD simulations although potentially at the expense of the accuracy (*32*).

Here, we show that by integrating SAXS and MD simulations based on the Martini coarse-grain force field (*34*, *35*) by means of metainference (*36*), we can efficiently generate an ensemble of structures representing the dynamics of linear polyubiquitins (Fig. 1). The ensembles allow the description of the dynamics of these complex systems at the single-residue level. Our results show how polyubiquitins can populate multiple conformational states but unexpectedly indicate that linear polyubiquitin chains and potentially polyubiquitins, in general, can be described by a simple self-avoiding polymer model. Various biophysical experiments are used to characterize the stoichiometry, kinetics, and thermodynamic properties of the binding of polyubiquitin to the NEMO_UBAN_ domain. Unexpectedly, our data demonstrate that NEMO_UBAN_ binds to di-, tri-, and tetraubiquitin (Ub_2_, Ub_3_, and Ub_4_) in all cases forming a 2:1 NEMO_UBAN_:Ub*_N_* complex in solution. Notably, a conformational ensemble for the NEMO_UBAN_:Ub_2_ complex rationalizes the 2:1 binding. Combined with our proposed polyubiquitin polymer model, this suggests how longer polyubiquitin chains may modulate NEMO recognition as well as bind more than one NEMO dimer.

## RESULTS

### A simple Martini modification improves the simulation of linear diubiquitin

**Fig. 2:**
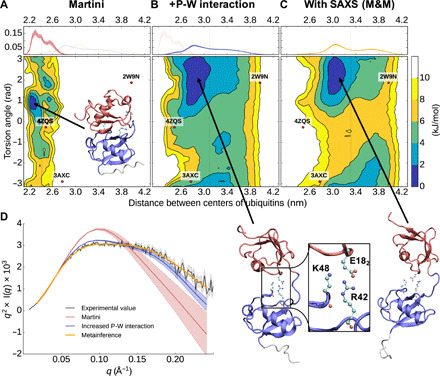
Characterization of the dynamics of linear diubiquitin. (**A** to **C**) Free energy landscapes (in kilojoules per mole) as a function of the distance between the center of mass of the two ubiquitin domains and their relative orientation (measured as the torsion angle between two axes defined using the first and second half of the sequence of each ubiquitin; see Methods). The dots represent the coordinates associated with the available diubiquitin crystal structures. On top is shown the probability distribution of the distance between the centers of the two ubiquitin domains. (**D**) Experimental and from-simulation calculated Kratky plot. The shaded area represents the error range.

We first evaluated the ability of the Martini coarse-grain force field to describe the dynamics of a linear Ub_2_. A metadynamics (*37*) simulation of Martini Ub_2_ resulted in an extremely compact ensemble of structures (Fig. 2A), which does not reproduce the measured SAXS intensities (Fig. 2D and fig. S1). In Fig. 2A, we report a free energy landscape (in kilojoules per mole) as a function of the distance between the centers of the two ubiquitin domains and their relative orientation; the average distance between the two domains is very short, around 2.41 ± 0.02 nm, with a preferential orientation of the two ubiquitin’s domain (measured as the torsion angle between two axes defined using the first and second half of the sequence of each ubiquitin; cf. Methods). The average radius of gyration of 1.73 ± 0.01 nm strongly underestimates the value of 2.23 ± 0.02 nm derived from SAXS (fig. S1). The ensemble seems to be able only to capture compact Ub_2_ configurations also when compared to the available crystal structures [PDB (Protein Data Bank) 2W9N (*38*) (open), 3AXC (*39*) (compact), and 4ZQS (*28*) (compact)]. This result is not unexpected for the Martini force field, and both weakening the protein-protein interactions (*40*, *41*) and increasing the protein-solvent interaction (*42*, *43*) have been suggested as possible solutions. A more complex water model, such as the Martini polarizable water (*44*), is also available, but at the expense of performance. Recent developments in atomistic force fields demonstrated the need for tuning solute-solvent interactions (*45*, *46*). Following recent approaches that have successfully improved atomistic and coarse-grained (CG) force fields, we repeated the same simulation after increasing by 5% the Martini water-protein Lennard-Jones interaction. This simple adjustment was sufficient to obtain a more expanded ensemble of structures as shown by the free energy landscape (Fig. 2B), without any additional computational cost (fig. S2). The new ensemble resulted in an improved, even if not yet quantitative, agreement with the SAXS data (Fig. 2D, blue curve, and fig. S1). The average distance between the domains increased to 3.10 ± 0.02 nm, and the protein can explore a much wider conformational space that now includes open and closed structures. In terms of the radius of gyration, the ensemble average resulted in 2.05 ± 0.01 nm to be compared with the 2.23 ± 0.02 nm derived from SAXS. Of note, comparing the free energy surface of the underdevelopment version of the Martini force field (Martini 3, currently in beta phase) with Martini 2.2 shows promising behavior by exploring more open conformation but may still benefit from increased protein-water (P-W) interaction (fig. S2). Nonetheless, our aim here is to obtain ensembles in quantitative agreement with the SAXS data without a large-scale force field reparameterization effort. This can be achieved at least, in principle, by integrating experimental information directly in the simulation by metainference (*36*). To show this, we run metadynamic metainference (M&M) (*47*) simulations (see Methods) with the K63-linked Ub_2_ and compared them with published atomistic ensembles (fig. S3). In Paissoni *et al.* (*48*), an ensemble of K63-linked Ub_2_ was generated through atomistic simulation with integrated SAXS data and validated against nuclear magnetic resonance (NMR) data. Overall, our energy landscape and the distribution of the radius of gyrations are comparable to the atomistic ones. The global minima region of the free energy landscape is correctly identified (red box in fig. S3), with a substantial improvement over an unrestraint atomistic ensemble (*48*). However, the modified Martini force field fails to correctly identify the conformation of very compact states (which are missing in the unrestrained atomistic ensemble). Comparing the contact maps shows that our approach still manages to identify the correct interdomain contact regions while being less specific. A weakening of the elastic network, which stabilizing the core of ubiquitin subunits (see Methods), or a further increase of the P-W interaction does not lead to improvement (fig. S4). A simple excluded volume model with integrated SAXS data is not sufficient to achieve qualitatively similar free energy surface (fig. S3). This shows that at least a qualitatively accurate description of the interdomain interactions is necessary to generate a precise SAXS ensemble.

Overall, our approach of coupling a modified Martini force field with SAXS manages to capture the overall balance between the compact and open states. It allows us to identify the global minima conformations and interdomain contact regions, but it might fail to capture specific compact states accurately. While a restrained atomistic simulation can lead to more accurate results, having a few hundred times faster sampling (fig. S2E) is a substantial benefit, making the sampling of more extended ubiquitin chains systems accessible.

### Metainference SAXS simulations of Martini linear diubiquitin quantitatively reproduce the experimental data

M&M (*47*) simulation (see Methods) for Martini linear Ub_2_ (including our modified water) result in an ensemble of configurations characterized by a flatter and broader free energy landscape (Fig. 2C) and in quantitative agreement with the experimental SAXS (Fig. 2, D and E, and fig. S1). With respect to the unrestrained simulation, the average distance between the two domains increased from 3.10 ± 0.02 nm to 3.32 ± 0.02 nm. The radius of gyration of the ensemble of 2.23 ± 0.01 nm quantitatively agrees with that derived from SAXS of 2.23 ± 0.02 nm. Qualitatively, the topology of the free energy landscape is comparable to the unrestrained simulation but translated to larger relative distances. Overall, the free energy landscape is quite flat with relatively limited free energy differences indicating that the two ubiquitin domains are relatively free to move with respect to each other. Therefore, Ub_2_ shows highly dynamical behavior, which cannot be described by a few individual structures. Instead, a full ensemble is required in agreement with previous findings on linear as well as other diubiquitins.

From the performance point of view, the SAXS on-the-fly calculation used by metainference is computationally demanding, but the use of a CG representation makes it far more affordable with respect to the same simulation performed at full atomistic resolution (fig. S2). The loss of performance resulting from the use of SAXS is justified by the increased accuracy of the resulting simulations. Note that it is not required to calculate the metainference SAXS restraint at every step of the simulation. By calculating it every five steps, we obtained a quantitatively equivalent ensemble at a fraction of the computational cost (fig. S2). Notably, using metainference allows us also to sample the scaling value, which is necessary to compare the experimental and computed SAXS curves. For Ub_2_, we observed a 3% higher scaling value for the simulation with increased P-W interaction and a 9% higher scaling value just with the Martini force field compared to the metainference solution (fig. S1).

### Linear polyubiquitin chains are preferentially extended, do not show long-range correlations, and can be described as self-avoiding polymers

**Fig. 3:**
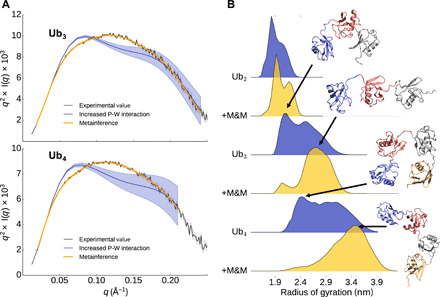
Characterization of the dynamics of linear tri- and tetraubiquitin. (**A**) Experimental and from-simulations calculated Kratky plot for tri- and tetraubiquitin. The shaded area represents the error range. (**B**) Distribution of the radius of gyration from the ensembles with and without M&M.

To investigate the dynamics of linear Ub_3_ and Ub_4_, we performed SAXS experiments on both proteins at different concentrations (fig. S5). The measured SAXS data were then used to perform M&M simulations (cf. Methods and table S1). In addition, unrestrained simulations based only on Martini with our modified water were also performed. In Fig. 3 (see also fig. S1), we show the comparison of the back-calculated SAXS with respect to the experimental measures of Ub_3_ and Ub_4_. The effect of our improved water diminishes for the longer polyubiquitin chains. A comparison of the radius of gyration for the Ub_2_, Ub_3_, and Ub_4_ ensembles show that while the unrestrained and restrained simulations sample a comparable range of compactness, the restrained simulations are shifted toward a more extended conformational space. The trend of the average radius of gyration (2.0, 2.7, and 3.3 nm for Ub_2_, Ub_3_, and Ub_4_, respectively; cf. Methods and fig. S1A) suggests an almost linear increase of the size of the protein with the number of ubiquitin monomers. The analysis of the free energy landscape for the Ub_2_ couples in Ub_3_ and Ub_4_ (fig. S6) qualitatively shows the same behavior, suggesting that the interdomain interactions are essentially only those between neighbor domains (i.e., between Ub_2_). For Ub_3_ and Ub_4_, this is confirmed by analyzing the free energy landscape of non-neighbor ubiquitin domains. Overall, the free energy landscape is flatter for larger polyubiquitin chains, indicating that the interaction with neighboring ubiquitin becomes less and less specific. Also, the distance (centers of the two ubiquitin domains) distribution shifts from a bimodal distribution for Ub_2_ to a flatter one for Ub_3_ and Ub_4_. We also observe that Ub_3_ samples more extended conformations for large distances >4.0 nm, while both Ub_4_ and Ub_3_ are forming more compact conformation below 2.6 nm. In fig. S7 (A to C), the free energy profiles are shown for the first-and-third ubiquitin Ub_3_(1-3) in Ub_3_ as well as for the first-and-third Ub_4_(1-3) and second-and-fourth Ub_4_(2-4). These landscapes are all qualitatively similar showing that the interaction between two non-neighbor ubiquitins is quite rare. The average distance between a 1-3 and 2-4 ubiquitin pair is around 6 nm, with an average angle of around 140°. The first ubiquitin does not influence the relative orientation of the third ubiquitin. The first-and-fourth Ub_4_(1-4) ubiquitin couple, as shown in fig. S7D, behaves similarly. Interactions between the first and fourth ubiquitin are also rare. In most cases, the distance between both ubiquitins is around 8.5 nm. There is also no strong preference for a specific torsion angle between all four ubiquitins.

**Fig. 4:**
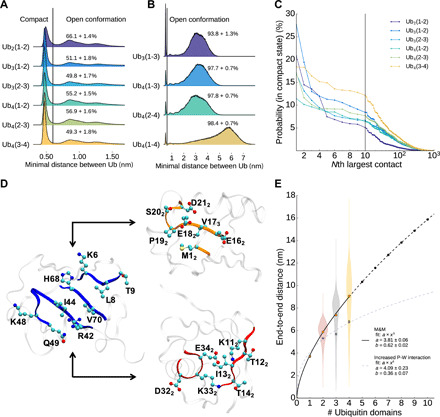
Intramolecular interactions of polyubiquitin. (**A**) Minimum distance distribution between two neighboring ubiquitin cores (residues 1 to 70, residues 77 to 146, and so forth). Structures with a minimal distance larger than 0.6 nm are defined as open. (**B**) Minimum distance distribution between two non-neighboring ubiquitin cores. Structures with a minimal distance larger than 0.6 nm are defined as open. (**C**) Probability of finding contacts between two amino acids of neighboring ubiquitin cores. (**D**) Interaction surface of two neighboring ubiquitins. Residues from the blue marked surface (first ubiquitin, left) are interacting with residues of the orange marked surface (middle) or red marked surface (right) of the second ubiquitin. (**E**) Average end-to-end distance of a linear polyubiquitin chain.

To further assess the presence of short and long-range interactions between neighbor and non-neighbor ubiquitin couples, we estimated the fraction of compact configurations by analyzing the minimum distance between neighbor and non-neighbor ubiquitin couples (Fig. 4, A and B). For neighbor and non-neighbor couples, there is a peak in the distribution around 0.5 nm. As already indicated by the free energy profiles, compact neighbor ubiquitin pairs represent around 40 to 50% of the ensemble, while contacts between non-neighbor couples are only present in around 8% for Ub_3_ and around 2% for Ub_4_, indicating an overall lack of compact states in linear polyubiquitins. A contact analysis for the Ub_2_ compact state indicates that this state is not structurally homogeneous. Even the most frequent contact is only present in 10 to 30% of all compact conformations, depending on the specific ubiquitin pair (Fig. 4C). On the other hand, even the 10th most frequent contact has still a probability between 5 and 15%, while the 100th most frequent one is still in the 1 to 5% range.

Nonetheless, all residues involved in the most frequent contacts belong to three distinct surfaces. These interactions define the preferred orientations between two adjacent ubiquitin pairs. All residues involved in the most frequent contacts from the first ubiquitin are on the same surface as the hydrophobic patch I44 (Fig. 4D), which is known to be also essential for interactions of ubiquitin with other proteins. The hydrophobic patch is also the main contributor for the interdomain contacts in the K63 Ub_2_ (fig. S3). This result is consistent between atomistic and coarse-grain simulation. The I44 surface interacts either with the surface around E18_2_ or I13_2_ (E18 or I13 would be the analog residues of the first ubiquitin). The E18_2_ surface is located opposite to the I44 surface, while the I13_2_ surface is roughly 90° rotated to the I44 and E18_2_ surface. In fig. S6 (H to M), the 10 most frequent contacts between ubiquitin cores for all adjacent ubiquitin pairs are illustrated. Ub_2_ is predominantly stabilized by salt bridges between the positive charged R42 and K48 and the negative charged E16_2_ and E18_2_. However, going to Ub_3_ and Ub_4_, electrostatic interactions become less important compared to van der Waals (vdW) interactions (fig. S7F). For Ub_2_, the Coulomb interaction between charged amino acids is responsible for 27% of the total interaction energy between two ubiquitin cores. This value goes as low as 9% for the Ub (3-4) pair of Ub_4_. Since the Martini force field has limitations in terms of electrostatics, the absolute ratios between vdW and Coulomb interactions are likely not meaningful. However, since our modified Martini force field with SAXS data manages to capture the important interaction regions, the relative changes between different ubiquitin pairs are likely qualitatively correct. The lower electrostatic interactions may also explain the flatter free energy surfaces of Ub_3_ and Ub_4_ neighbor pairs (fig. S4). On the other hand, the increased role of vdW interactions in Ub_3_ and Ub_4_ is compatible with the increase in the compact population of diubiquitin couples. Last, while in Ub_2_ the I44 surface prefers to interact with the E18_2_ surface, interactions between the I44 and the I13_2_ surface are more important for the last pair of Ub_4,_ causing a shift of the preferred orientation between both ubiquitins.

Overall, our linear Ub_2_, Ub_3_, and Ub_4_ ensembles indicate that linear polyubiquitins are extended polymers, whose dynamics are mostly uncorrelated over a distance of more than one ubiquitin domain. In Fig. 4E, this behavior is further highlighted by plotting the end-to-end distance as a function of the number *N* of ubiquitin domain, *e*2*e*(*N*). Fitting the data with a power law (including the end-to-end distance for Ub_1_) resulted in *e*2*e*(*N*) = 3.81*N*^0.62^ in remarkable agreement with Flory theory for self-avoiding polymers (*49*). This is remarkable, since generally, proteins do not behave as self-avoiding chain, showing also less entropy in the denatured state. This behavior is not shown in simulations just with increased P-W interactions, which have a substantially lower exponent of 0.36. It is tempting to speculate that all polyubiquitins may be described as self-avoiding polymers following the same relationship for the end-to-end distance but with a different prefactor (i.e., characteristic length) associated with the distance between the C-terminal glycine and the specific linkage side chain. For K63-linked polyubiquitins, we could test this, even if only with the monoubiquitin and diubiquitin, making use of a SAXS-based ensemble that we recently published (*48*). By fixing the exponent to 0.6 and setting the prefactor to the average distance between the C terminus and K63, the expected *e2e* distance of Ub_2_ fits the ensemble. This suggests that the fit can be used to predict the behavior for longer K63-linked Ub*_N_* chains (fig. S6). Extrapolating for other linkages, we observe that K11- and K48-linked polyubiquitin, which are known to populate more compact states in solution (*50*, *51*), have a shorter distance between the C terminus and the lysine and would consequently show smaller prefactors and populate systematically more compact states than K63- and M1-linked.

### The conformational entropy of long linear polyubiquitins modulates NEMO binding

To study the dynamics and changes of linear polyubiquitin dynamics upon binding to cognate proteins, we used simulations and experiments to characterize the interaction of the NEMO UBAN domain (NEMO_258–350_) to the linear polyubiquitins Ub_2_, Ub_3_, and Ub_4_. The NEMO UBAN domain is a dimer in solution (*23*). Previous studies have shown two different binding stoichiometries in solution and crystalline state for Ub_2_: with either two NEMO monomers bound to one Ub_2_(2:1) or two NEMO monomers bound to two Ub_2_ (2:2) (*23*, *24*). Polyubiquitin chains longer than Ub_2_ harbor potentially more than one binding site and could thus bind more than one NEMO dimer with a theoretical stoichiometry for Ub*_N_* of 2(*N* − 1):1 (NEMO:Ub*_N_*). A crystal structure (PDB 5H07) shows the binding of two linear Ub_3_ to four ABIN monomers (a homolog of NEMO that also forms a dimer in solution) (*52*). The observed binding mode requires Ub_3_ to be in a relatively compact and univocally oriented configuration to avoid steric hindrances between the two ABIN dimers. This would markedly decrease the entropy not only of each diubiquitin couple but also that of the overall chain and thus should be entropically disfavored in solution (fig. S7). Isothermal titration calorimetry (ITC) experiments show that in solution, only one ABIN dimer binds to Ub_3_ (*52*), arguing that higher stoichiometries are artifactual, induced by crystal packing, and do not reflect the solution assembly.

**Fig. 5:**
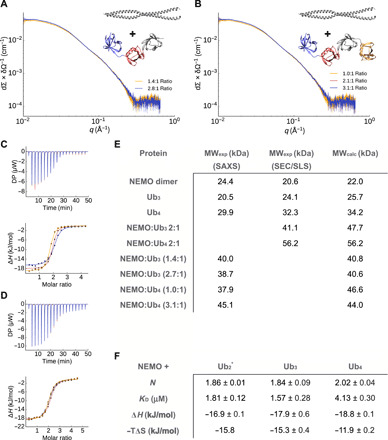
Effect of chain length on the binding of NEMO. (**A**) and (**B**) SAXS experiments for different ratios of NEMO and Ub_3_ (A) and Ub_4_ (B). (**C** and **D**) ITC measurement of the interaction of NEMO with Ub_3_ (C) and Ub_4_ (D). NEMO was titrated into the polyubiquitin solutions. The experiment was repeated three times. DP, differential power. (**E**) MW determination. SAXS and SEC in combination with SLS were used to determine the MW of NEMO, Ub_3_, Ub_4_, NEMO:Ub_3_, and NEMO:Ub_4_. The conditions were 50 mM tris HCl (pH 8) and 300 mM NaCl. (**F**) ITC measurement of the NEMO interaction with Ub_2_, Ub_3_, and Ub_4_. NEMO was titrated into the ubiquitin solutions in 50 mM sodium phosphate (pH 7) and 50 mM NaCl. Values are averages ± SEs from three measurements. The individual ITC curves are shown in fig. S7. *Experiments taken from Vincendeau *et al.* (*23*). A stoichiometry of *N* = 2 corresponds to one NEMO dimer binding to one polyubiquitin protein.

To characterize the binding in solution of NEMO to Ub_3_ and Ub_4_, we performed SAXS (Fig. 5 and fig. S5), ITC (Fig. 5 and fig. S9), size exclusion chromatography (SEC) coupled with static light scattering (SLS) (Fig. 5, fig. S9, and table S2), and surface plasmon resonance (SPR) (fig. S9). Extending Ub_2_ to Ub_3_ and Ub_4_ does not affect the (2:1) binding stoichiometry, with either polyubiquitin protein binding two NEMO monomers (one NEMO dimer) (Fig. 5). In SEC experiments with SLS, we detected NEMO:Ub_3_ complexes with molecular weights (MWs) ranging from 41 to 45 kDa, which is similar to the MW of the calculated 2:1 NEMO:Ub_3_ complex (47.7 kDa), while for NEMO:Ub_4_ complexes only a single peak is found with MW between 53 and 56 kDa (calculated MW 2:1 NEMO:Ub_4_, 56.2 kDa). SAXS measurements confirmed the stoichiometry observed by SLS-SEC. ITC indicates that NEMO binds to Ub_3_ and Ub_4_ with very similar enthalpy (Δ*H* of −17.9 and −18.8 kJ/mol, respectively), suggesting that the molecular interactions and binding interfaces between NEMO and the different polyubiquitins are similar to the one described for NEMO:Ub_2_ (*23*) (Δ*H* of −16.9 kJ/mol). Affinities for Ub_3_ and Ub_4_ are 1.6 and 4.1 μM close to 1.8 μM obtained for Ub_2._ ITC data comparing shorter and longer polyubiquitins may suggest that longer polyubiquitins can form long-range, flanking interactions with NEMO, resulting in a gain of enthalpy and loss of entropy with respect to shorter ones. SPR confirmed the binding between NEMO and Ub_3_ and Ub_4_ with equilibrium dissociation constants (*K*_D_) of 9.6 and 6.4 μM for Ub_3_ and Ub_4,_ respectively.

**Fig. 6:**
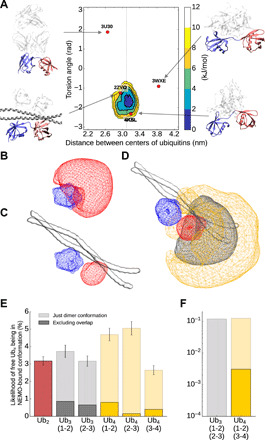
Comparison between free and NEMO-bound polyubiquitin ensembles. (**A**) Free energy landscapes (in kJ/mol) as a function of the distance between the centers of the two ubiquitin domains and their relative orientation for Ub_2_ bound to NEMO. The dots represent the coordinates associated with the available crystal structures with Ub_2_ bound to different proteins. (**B** and **C**) Conformational space of free (B) and NEMO-bound (C) ubiquitin pairs in Ub_2_. The blue area represents the first ubiquitin, while the red area shows the conformation of the second ubiquitin relative to the first one. (**D**) Conformational space of third (gray area) and forth (orange area) ubiquitin in Ub_4_ with the first Ub pair being in a NEMO-bound conformation. (**E** and **F**) Probability of free Ub_2_, Ub_3_, and Ub_4_ of being in a NEMO-bound conformation for one (E) or two (F) NEMO dimers. The transparent bars show the likelihood of the individual pairs being in the NEMO-bound conformation (root mean square deviation <6 A compared to the average Ub_2_ structure in the NEMO-bound simulation). The dark bars show the probability of being in the NEMO-bound conformation, excluding structures with an overlap between the nonbound ubiquitins and NEMO.

To explain the contradictory observation of the stoichiometry in solution and in crystal and to better understand the molecular recognition between linear polyubiquitins and NEMO, we characterized the dynamics of a NEMO_258–350_:Ub_2_ complex. An M&M Martini simulation was performed including SAXS data for the complex previously measured (Fig. 6A and fig. S10). The resulting ensemble of structures highlights how binding to NEMO strongly decreases the conformational freedom of linear Ub_2_ (Figs. 2 and 6A). Neither the ensemble nor the crystal structures of other bound Ub_2_ are located close to the minima of the free Ub_2_ ensemble, which has a different distance and orientation between both ubiquitins. Ub_2_ residues building up the NEMO_258–350_:Ub_2_ interface overlap with those involved in the interdomain interactions (fig. S10), in particular residues around the hydrophobic patch I44 and the previously mentioned E18_2_ surface. The observed interaction sites are in agreement with observed NMR chemical shifts perturbations reported in Vincendeau *et al.* (*23*). The ensemble also provides a possible explanation for the different binding stoichiometry observed in solution (NEMO:Ub_2_ 2:1) and crystalline state (NEMO:Ub_2_ 2:2). A detailed analysis of the NEMO-binding sites indicates that almost all the residues of the NEMO unoccupied site are less exposed to the solvent than those on the occupied one (fig. S10H). While the solvent accessible surface area of the occupied site is 13 nm^2^, the one of the unoccupied is 11.9 nm^2^, showing that both binding sites are not equal after the binding of Ub_2_. Together, these observations indicate that, in solution, binding of linear Ub_2_ to NEMO_UBAN_ induces allosteric effects that modulate the overall structure and dynamics of the NEMO dimer. These observations also suggest that the 2:2 highly symmetric binding mode observed in the dense and ordered crystalline state becomes entropically unfavorable to the more flexible and far less dense solution state of the complex.

## DISCUSSION

Structural biology investigations on polyubiquitins have mostly focused on diubiquitins, observing that different protein linkages correspond to different protein dynamics leading to different exposed regions for the binding with partners (*48*, *50*, *51*, *53*–*59*). Ubiquitin signaling has been found associated not only to the linkage type but also to the length of the ubiquitin chains (*8*–*10*). Here, we first develop an efficient and accurate integrative approach to characterize the conformational ensembles of linear polyubiquitin by combining the Martini coarse-grain force field with SAXS experiments in the framework of metainference. We then use our method to try to rationalize the length-dependent behavior of linear polyubiquitins and the consequence for the interaction with their partner NEMO. Figure 6 rationalizes the observed differences in binding by comparing our free Ub*_N_* ensembles with our NEMO-bound ensemble. The fraction of bound-like configurations in the Ub_2_ ensemble is a small fraction of the total ensemble, suggesting a large conformational entropy loss upon binding. This is likely compensated by a release of a large number of water molecules from the binding interfaces upon binding to result in a final entropy gain as indicated by ITC (Fig. 5). The probability of finding at least one diubiquitin pair in a bound-like configuration in the Ub_3_ and Ub_4_ ensemble increases slightly more than linearly (3.2, 6.7, and 12.0% for Ub_2_, Ub_3_, and Ub_4_, respectively), suggesting that longer polyubiquitins are likely to more favorably bind NEMO with respect to shorter ones. The SEC-SLS experiments show that NEMO bound to Ub_4_ eluted as a single bound peak in comparison with Ub_3_ (table S2 and fig. S9, D and E). Since both NEMO:Ub_4_ and NEMO:Ub_3_ have similar *K*_D_s, this can indicate a difference in kinetic stability. To provide a structural interpretation for this hypothesis, we calculated the actual probability of finding the full polyubiquitin in a configuration compatible with the binding (and thus avoiding configurations that would lead to a steric clash with NEMO (Fig. 6, B to D) from our free polyubiquitin ensembles. The probability decreases from Ub_2_ to Ub_4_ (3.2, 1.5, and 1.4% for Ub_2_ Ub_3,_ and Ub_4_, respectively), which can lead to entropy loss. At the same time, nonspecific flanking interactions between polyubiquitin and NEMO far from the binding site can increase the enthalpy. This is also in agreement with previous measures where, using a longer NEMO construct (NEMO_242–419_) that could provide more surface for interactions, affinities of 3 and 0.3 μM were reported for Ub_2_ and Ub_4_, respectively (*60*). This principle is also common for intrinsically disordered proteins possibly modulating the lifetime of complexes (*61*). These long-range effects would be less pronounced for a less entropic chain and can play a length-dependent role in the overall interaction.

The probability of finding two diubiquitin pairs in a bound-like configuration is essentially negligible for both Ub_3_ and Ub_4_ providing a rationale why a 2:1 NEMO_258–350_:Ub_4_ interaction is favored by entropy with respect to the 4:1. Making use of our polymer model, we can also speculate that a long-enough polyubiquitin may be able to bind two NEMO dimers with a higher-order (i.e., 4:1) stoichiometry with respect to the 2:1 observed for the Ub_2_ to Ub_4_ range. Given that the end-to-end distance of Ub_2_ corresponds to one-half of that of our NEMO construct (5.8 nm for Ub_2_ and 11.4 nm for NEMO), and that the two Ub_2_ units that bind the two NEMO should be allowed to be flexible, one can estimate the length of this polyubiquitin to be such that *e*2*e*(*N* − 2) ≥ 11.4 nm. This results in a minimum length of *N* *= 8* ubiquitins. While this result will not be quantitative when considering a full-length NEMO, it suggests a possible need for these long chains in the assembly of the IKK complex.

### Conclusions

The combined use of experiments and MD simulation is a powerful tool to investigate the structure and dynamics of biomolecules and provide a ground for the functional interpretation of protein dynamics. Here, we combined SAXS and CG Martini simulations to accurately and efficiently study the conformational dynamics of linear polyubiquitins and their binding to NEMO. The resulting conformational ensembles allowed us to propose that linear polyubiquitin behave as self-avoiding polymer chains. This might also apply for polyubiquitins in general (with different characteristic lengths). Combining structural studies with multiple biophysical experiments, we provide a systematic assessment of the effect of the polyubiquitin chain length in the molecular recognition of cognate proteins, suggesting that polyubiquitin may modulate the binding with their partners in a length-dependent manner.

## METHODS

### CG MD simulations

CG MD (CG-MD) simulations were applied to investigate the dynamic of linear di-, tri-, and tetraubiquitin (Ub_2_, Ub_3_, and Ub_4_), as well as Ub_2_ with bound NEMO. In total, 11 different simulations have been performed with a total simulation length of 780 μs. An overview of all simulations can be found in table S1.

All CG simulations were run using Gromacs 2016.3 (*62*) and the Martini force field (*34*, *35*). In addition, an elastic network model with a force constant of 500 kJ mol^−1^ nm^−2^ was used to conserve the secondary and tertiary structure (*34*, *63*). In the case of polyubiquitin, the elastic network inside was only defined for the backbone beads of the core region (from residues 1 to 70, 77 to 146, 153 to 222, or 229 to 298) and not between different domains nor for the linker region. All simulations were performed with periodic boundary conditions, and the systems were solvated with a 0.1 M NaCl solution and run as an isothermal-isobaric (NpT) with a temperature of 300 K and a pressure of 1 bar using 20-fs time steps. To control the temperature and pressure, the v-rescale thermostat (*64*) was used with a coupling constant τ_t_ = 1.0 ps together with the Parrinello-Rahman barostat (*65*) and a coupling constant of τ_p_ = 20.0 ps and compressibility of χ = 3.0 × 10^−4^ bar^−1^. Nonbonded interactions were treated with a dielectric constant of 15 and using a cutoff distance of 1.1 nm. Visual Molecular Dynamics (VMD) was used for visualization (*66*).

For many simulations, the Martini 2.2. force field was modified to increase the P-W interaction, which was achieved by giving water beads their own atom type with a 5% larger C6 parameter in interactions with all other atom types, resulting in around 5% higher P-W interactions.

Parallel-biased metadynamics (*37*, *67*), as implemented in PLUMED2 (*68*), was used to enhance the sampling of the conformational space, together with the multiple-walker approach (*69*) with 112 replicas for free polyubiquitin or 64 replicas for free NEMO as well as bound NEMO with Ub_2_, where each replica had a different starting conformation. The used collective variables were the distances between the centers of the different ubiquitin cores, a torsion angle between the centers of residues 1 to 36 and 37 to 70 of two different ubiquitins, the radius of gyration (calculated only with backbone atoms) and the alphabeta collective variable describing the torsional angles for linkers between the ubiquitin pairs. In total, 4 collective variables for Ub_2_, 9 for Ub_3_, and 16 for Ub_4_ were used. In the case of simulations with NEMO, additional collective variables were used for the shape of NEMO and distances between Ub_2_ and NEMO. The bias factor of the well-tempered metadynamics (*70*) was set to 10, the frequency for the hill addition was 200 (every 4 ps), the height of Gaussian hills was 0.1 kJ/mol for simulations with Ub_2_ and Ub_3_, 0.075 kJ/mol for simulations with tetraubiquitin and 0.02 kJ/mol for the simulation with NEMO bound to Ub_2_. The flexible Gaussian approach (*71*) was used to determine the Gaussian width during the simulation.

Metainference (*36*), a method based on Bayesian inference, was used to integrate experimental SAXS data into simulations and was coupled with metadynamics (M&M) (*47*). The calculation of the SAXS intensities from a CG Martini representation is implemented in the PLUMED-ISDB module (*72*, *73*) using the parameters derived by Niebling *et al.* (*74*) and the Debye equation. The SAXS data of the different systems were fitted with a 16th-degree polynomial to calculate points used for restraints. Twenty-one equidistant points for *q* between 0.017 and 0.24 nm^−1^ were used for Ub_2_ and Ub_3_ and for free and bound NEMO, 19 points for *q* between 0.025 and 0.19 nm^−1^ for Ub_4_. The rage depends on the quality of the experimental data. An initial scaling value was determent by comparing the calculated and experimental SAXS intensities for the lowest *q* value. Metainference was used with the outlier noise model (*36*) for each data point, and the restraints were applied every fifth step. A scaling faction and offset for the experimental data were sampled using a flat prior between 0.9 and 1.1 or −1 and 1. The error for calculating an average quantity σ_mean_ was determined automatically (*75*) from the maximum SE over 2 ps of simulations.

Six different simulations with Ub_2_ were performed using: Martini 2.2 with metadynamics, with increased P-W interaction and metadynamics, with M&M, with M&M applied every step, and with Martini 3 beta. Notably, Martini 3 beta was not stable with metadynamics; therefore, 112 replicas were run on a longer time scale. The SAXS data of Ub_2_ were taken from Vincendeau *et al.* (*23*). Notably, the profile was measured with Ub_2_ containing a His-tag on the N terminus that was also modeled. Ub_3_ and Ub_4_ simulations were run with increased P-W interaction, metadynamics with and without metainference, and SAXS. All polyubiquitin simulations were run for at least 500 ns per replica. The simulations with free NEMO and NEMO bound to Ub_2_ were performed with increased P-W interaction and M&M with SAXS for at least 100 ns per replica. The SAXS data of NEMO bound to Ub_2_ were taken from Vincendeau *et al.* (*23*).

Five simulations were performed with a K63 Ub_2_ construct, using Martini 2.2 with +5% increased P-W interaction and metadynamics and with +5% increased P-W interactions and M&M, as well as two additional tests with +10% increased P-W interactions and a weaker elastic network with a force constant of 500 kJ mol^−1^ nm^−2^, using the same SAXS data and constructs as described in Paissoni *et al.* (*48*). In addition, two control simulations with K63 Ub_2_ were performed using a Martini bead–based excluding volume. The Martini nonbonded interactions were replaced with repulsion term. The plumed input files, as well as the modified Martini topology files, are deposited in PLUMED-NEST (*76*) as plumID:20.009.

### Protein expression and purification

Human NEMO_258–350_ C347S was expressed and purified as described in Vincendeau *et al.* (*23*). Protein concentration was determined by measuring the absorbance at 205 nm using specific absorbance for NEMO_258–350_ C347S of 300,990 M^−1^ cm^−1^, respectively (*77*).

The constructs for the expression of Ub_3_ and Ub_4_ were a gift of P. Elliott and D. Komander [Medical Research Council (MRC) Laboratory of Molecular Biology, Cambridge, UK]. The constructs were transformed into *Escherichia coli* strain BL21 (DE3) and cultured at 20°C in 2-liter flasks containing 500 ml of ZYM 5052 autoinduction medium (*78*) and carbenicillin (100 μg/ml). Cells were harvested by centrifugation after reaching saturation, resuspended in 60-ml lysis buffer [50 mM tris-HCl, 300 mM NaCl, 10 mM MgCl_2_, deoxyribonuclease I (10 μg/ml), 1 mM AEBSF.HCl (4-(2-Aminoethyl)benzenesulfonyl fluoride hydrochloride), 0.2% (v/v) NP-40, and lysozyme (1 mg/ml; pH 8.0)], and lysed by sonication. The lysate was clarified by centrifugation (40,000*g*) and filtration (0.2 μM). The supernatant was heated in a water bath for 10 to 15 min at 60°C and the precipitate removed by centrifugation. The supernatant was dialyzed overnight against 2 liters of buffer A [50 mM sodium acetate (pH 4.5)], clarified by centrifugation and applied to a 5-ml HiTrap SP HP column (GE Healthcare), and equilibrated in buffer A. Bound proteins were eluted using a linear gradient (10 column volumes) from 0 to 1 M NaCl in buffer A using an Äkta Purifier (GE Healthcare). Elution fractions (1.6 ml) were collected in wells containing 250 μl of 1 M tris-HCl (pH 9.0). Fractions containing Ub_3_ or Ub_4_ were pooled, concentrated and applied to a HiLoad 16/600 Superdex 75 column (GE Healthcare), and equilibrated in buffer B [50 mM tris-HCl and 100 mM NaCl (pH 7.4)]. The main elution peak containing Ub_3_ or Ub_4_ was collected and concentrated to approximately 3 to 6 mg/ml, flash-frozen, and stored at −80°C. Protein concentrations were determined by measuring the absorbance at 205 nm using specific absorbance for Ub_3_ and Ub_4_ of 747,790 and 997,980 M^−1^ cm^−1^, respectively (*77*). The Ub_4_ concentration values used on interaction studies of Ub_4_ with NEMO were corrected by 30% on the basis of the SEC with SLS results.

### Small-angle x-ray scattering measurements

SAXS measurements were performed on a Rigaku BioSAXS-1000 instrument with an HF007 microfocus generator equipped with a Cu target at 40 kV and 30 mA. Transmissions were measured with a photodiode beamstop, and *q* calibration was made by an Ag-behenate measurement. Absolute calibration was done with calibrated glassy carbon (*79*). Measurements were done in four 900-s frames, which were averaged. Under these conditions, no radiation damage was detected. Circular averaging and background subtraction were done with the Rigaku SAXSLab software v 3.0.1r1.

Radii of gyration were calculated with the ATSAS package v 2.8.0 (*80*). Fits for the MW determination were made in Origin v 9. SAXS measurements were made at 293 K using a buffer containing 300 mM NaCl and 50 mM tris-HCl at pH 8.0. Experiments on the free proteins were performed at the following concentrations (fig. S3, I and J): NEMO at 2.34, 4.62, and 7.72 mg/ml; Ub_3_ at 3.41, 6.72, and 11.17 mg/ml; and Ub_4_ at 4.5, 9.1 and 15.1 mg/ml. Experiments with NEMO and Ub_3_ and Ub_4_ at different ratios were performed at two concentrations (between 3 and 4 and 7 and 8 mg/ml) at the following ratios: NEMO:Ub_3_ at 1.4:1 and 2.7:1 ratios and NEMO:Ub_4_ at 1.0:1, 2.1:1, and 3.1:1 ratios. No concentration-dependent effects were detected.

### Isothermal titration calorimetry

ITC measurements were carried out at 298 K using a PEAQ-ITC titration microcalorimeter (MicroCal, Malvern). The NEMO-to-Ub_3_ calorimetric titration consisted of 19 injections of 2 μl of a 2.13 mM NEMO solution, into the reaction cell containing 300 μl of 94.71 μM Ub_3_, at a stirring speed of 750 rpm. The NEMO-to-Ub_4_ calorimetric titration consisted of 19 injections of 2 μl of a 2.84 mM NEMO solution, into the reaction cell containing 300 μl of 120.6 μM Ub_4_, at a stirring speed of 750 rpm. Sample conditions were 50 mM sodium phosphate (pH 7.0) and 50 mM NaCl. The heat of dilution was obtained by titrating NEMO into the sample cell containing only buffer. Experiments were done in triplicate. The ITC data were analyzed using the software MicroCal PEAQ-ITC analysis software. Parameters are presented as averages ± SEs.

### SEC with SLS

SLS experiments were performed of NEMO mutant (C347S) in complex with tri- and tetraubiquitin at 30°C using a Viscotek TDA 305 triple array detector (Malvern Instruments) downstream to an Äkta Purifier (GE Healthcare) equipped with an analytical size exclusion column (Superdex 200 10/300 GL, GE Healthcare) at 4°C. The samples were run at approximately 8 mg/ml at a flow rate of 0.5 ml/min. The experiments were performed using a tris buffer [50 mM tris-HCl and 300 mM NaCl (pH 8.0)] and a phosphate buffer [50 mM sodium phosphate and 50 mM NaCl (pH 7.0)]. The molecular masses of the samples were calculated from the refractive index and right-angle light-scattering signals using Omnisec (Malvern Instruments). The SLS detector was calibrated with a bovine serum albumin (BSA) solution (4 mg/ml) using 66.4 kDa for the BSA monomer and a *dn/dc* value of 0.185 ml/g for all protein samples.

### SPR measurements

SPR measurements were performed at 25°C using a Pioneer FE instrument (FortéBio, Molecular Devices). Ub_3_ and Ub_4_ were covalently immobilized onto two different flow cell channels on a biosensor chip by amine coupling to 456 and 721 response unit, respectively, using a 10 mM NaOAc (pH 5) immobilization buffer. NEMO was injected in a twofold concentration series over immobilized ubiquitins at a flow rate of 30 μl/min using a phosphate-buffered saline running buffer [50 mM sodium phosphate, 50 mM NaCl, and 0.005% Tween 20 (pH 7)]. The data were analyzed using Qdat Data Analysis Tool version 2.6.3.0 (FortéBio). The sensorgrams were corrected for buffer effects and unspecific binding to the chip matrix by subtraction of blank and reference surface (a blank flow cell channel activated by injection of EDC/NHS (N-ethyl-N′-(3-dimethylaminopropyl)carbodiimide hydrochloride/N-hydroxysuccinimide) and inactivated by injection of ethanolamine). The equilibrium dissociation constants (*K*_D_) were estimated by plotting responses at equilibrium (Req) against the injected concentration and curve fitted to a Langmuir (1:1) binding isotherm.

## Supplementary Material

abc3786_SM.pdf

## SUPPLEMENTARY MATERIALS

Supplementary material for this article is available at http://advances.sciencemag.org/cgi/content/full/6/42/eabc3786/DC1

## Appendix

View/request a protocol for this paper from *Bio-protocol*.
