# Supplementary material for: The dynamics of linear polyubiquitin
Source: Sci Adv. 2020 Oct 14;6(42):eabc3786. doi: 10.1126/sciadv.abc3786 (PMC7556843; doi:10.1126/sciadv.abc3786)
Supplement: abc3786_SM.pdf [file abc3786_SM.pdf]

Science Advances
AAAS

advances.sciencemag.org/cgi/content/full/6/42/eabc3786/DC1

Supplementary Materials for

The dynamics of linear polyubiquitin

Alexander Jussupow, Ana C. Messias, Ralf Stehle, Arie Geerlof, Sara M. Ø. Solbak, Cristina Paissoni, Anders Bach, Michael Sattler*, Carlo Camilloni*

*Corresponding author. Email: sattler@helmholtz-muenchen.de (M.S.), carlo.camilloni@unimi.it (C.C.)

Published 14 October 2020, *Sci. Adv.* **6**, eabc3786 (2020)
DOI: 10.1126/sciadv.abc3786

This PDF file includes:

Figs. S1 to S10
Tables S1 and S2

A	Scaling value	Offset	Chi-Square	R_g	R_g with expression tag
Di-ubiquitin					
**Martini**	1.093 +- 0.037	0.036 +- 0.018	0.3575	1.654 +- 0.005	1.729 +- 0.004
**Increased P-W interaction**	1.034 +- 0.010	0.151 +- 0.005	0.0334	1.933 +- 0.006	2.050 +- 0.006
**With SAXS**	1.003 +- 0.001	0.005 +- 0.0004	0.0001	2.034 +- 0.006	2.230 +- 0.006
		Experimental Value:			2.23 +- 0.02
Tri-ubiquitin					
**Increased P-W interaction**	1.042 +- 0.013	0.015 +- 0.023	0.0695	2.417 +- 0.013	
**With SAXS**	0.990 +- 0.001	0.021 +- 0.001	0.0002	2.699 +- 0.013	
		Experimental Value:		2.66 +- 0.02	
Tetra-ubiquitin					
**Increased P-W interaction**	1.079 +- 0.019	0.008 +- 0.037	0.1241	2.851 +- 0.022	
**With SAXS**	0.983 +- 0.0003	0.032 +- 0.001	< 0.0001	3.332 +- 0.018	
		Experimental Value:		3.301 +- 0.04	

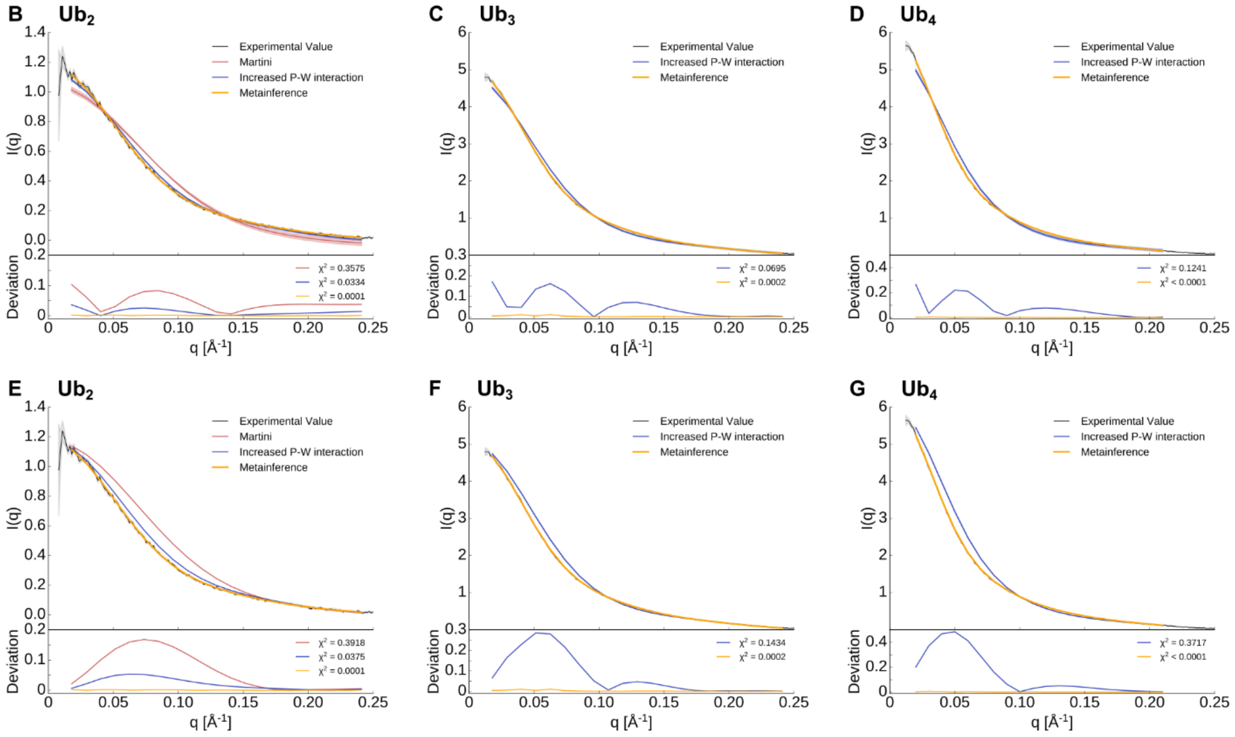

Fig. S1. Experimental and theoretical SAXS curves of poly-ubiquitin. A) Comparison of the quality of the SAXS calculations for different simulation setups. **B-D)** SAXS intensities obtained from simulations fitted to the experimental curves of Ub_2, Ub_3, and Ub_4. **E-G)** SAXS intensities obtained from simulation with the scaling factor obtained by the M&M simulation. The scaling value should be similar for all structures and ensembles of the same protein since the

SAXS intensities for scattering vectors close to q = 0 $Å^{-1}$ are mostly independent of the structure and dynamics of the system. In many cases, the SAXS curve calculated directly from a given structure or ensemble is simply fitted to the experimental data (B-D). This approach produces scaling values that are generally not transferable between different ensembles. Using the same scaling value (E-G) leads to a reduction of the agreement between theoretical and experimental SAXS profiles for non-Metainference ensembles.

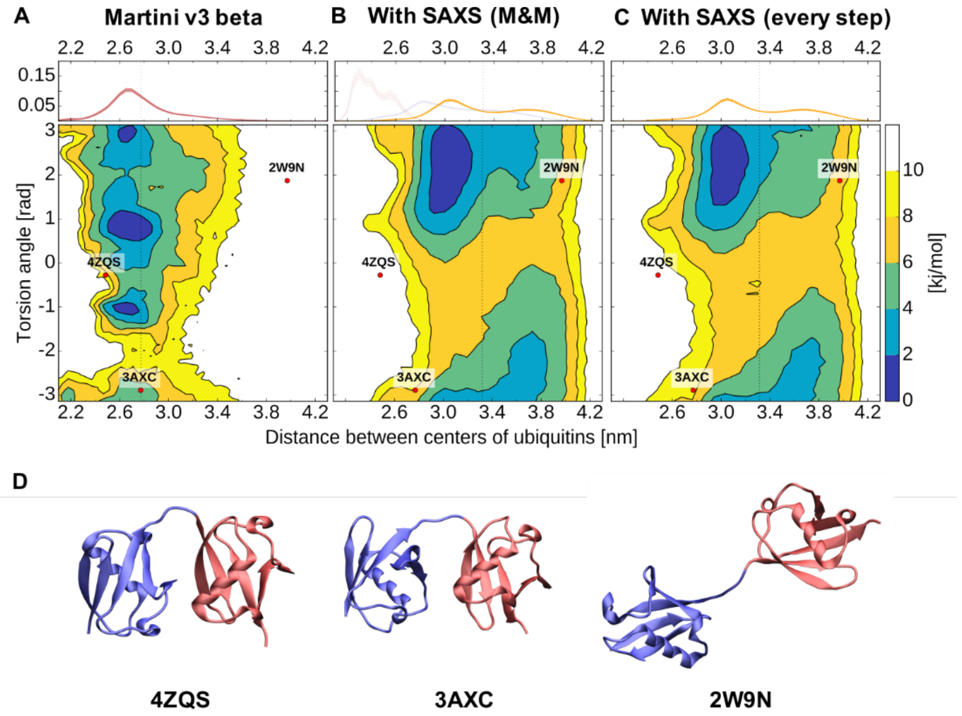

E

Cores per replica	1	2	4	8	16
Di-ubiquitin					
Martini	415	730	1296	2098	3228
Martini + Metadynamics	352	564	928	1292	1590
With SAXS	142	255	466	740	1088
With SAXS - every step	40	78	149	254	412
Atomistic	< 1	1	2	3	6
Tri-ubiquitin					
With SAXS	98	182	329	541	816
Tetra-ubiquitin					
With SAXS	51	95	178	313	485

Fig. S2. Free energy surfaces of linear di-ubiquitin for different simulation setups. A-C) Free energy landscapes (in kJ/mol) as a function of the distance between the centers of the two ubiquitin domains and their relative orientation. The dots represent the coordinates associated with the available di-ubiquitin crystal structures. On top is shown the probability distribution of the distance between the centers of the two ubiquitin domains. **D)** Crystal structures of free Ub_2. **E)** Performance for the different simulation setups. All values are in ns/day.

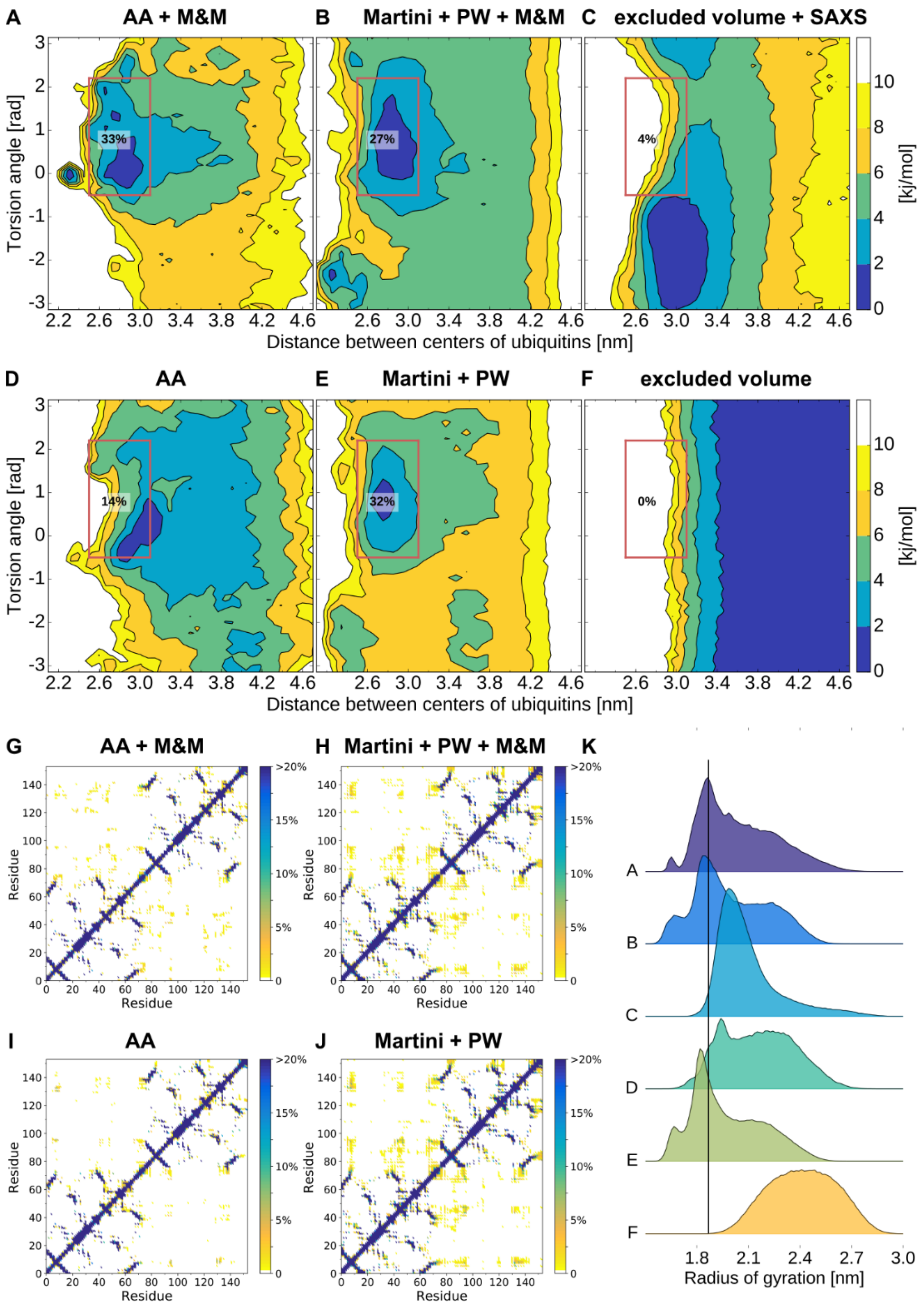

Fig. S3. Comparison between atomistic (AA), coarse-grain (Martini) and an excluded volume ensemble for K63 Ub$_2$. A-F) Free energy landscapes (in kJ/mol) as a function of the distance between the centers of the two ubiquitin domains and their relative orientation. The value in the red box represents the amount of conformations inside the boundaries. **G-J)** Contact maps of atomistic (G,H) and coarse-grain (I,J) ensembles. **K)** Distribution of the radius of gyration from the atomistic, coarse-grain and self-avoiding ensembles with and without including of SAXS data.

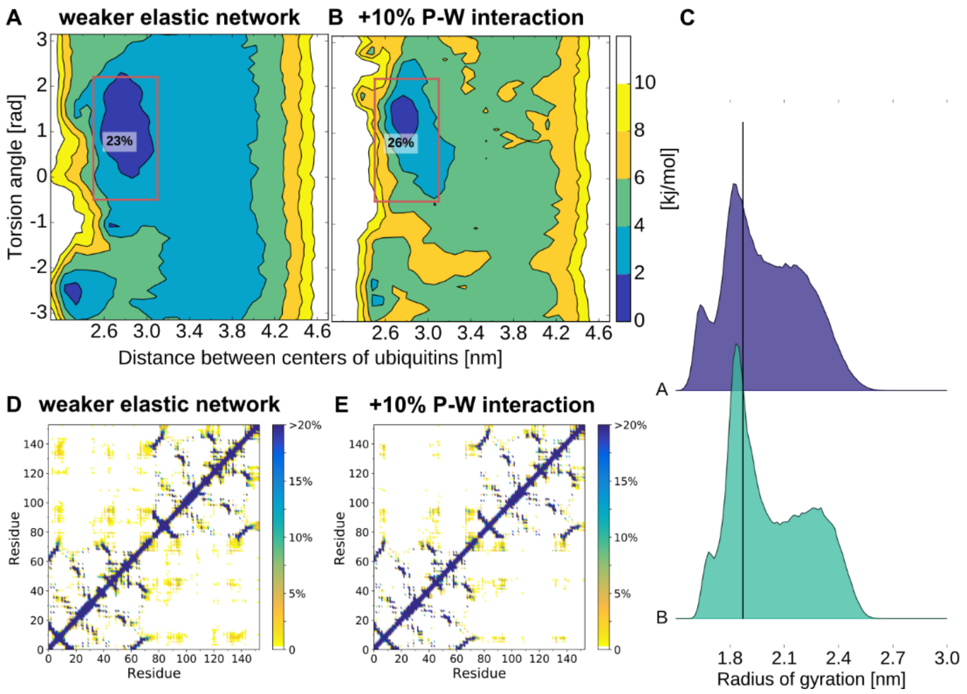

Fig. S4. Effect of weaker elastic network and +10% increased protein-water (P-W) interaction on the K63 Ub_2 ensemble. A-B) Free energy landscapes (in kJ/mol) as a function of the distance between the centers of the two ubiquitin domains and their relative orientation. The value in the red box represents the amount of conformations inside the boundaries. **C)** Distribution of the radius of gyration from the ensemble with weaker elastic network and +10% increased protein water interaction. **D-E)** Contact maps of ensembles with weaker elastic network (D) and +10% increased protein water interaction (E).

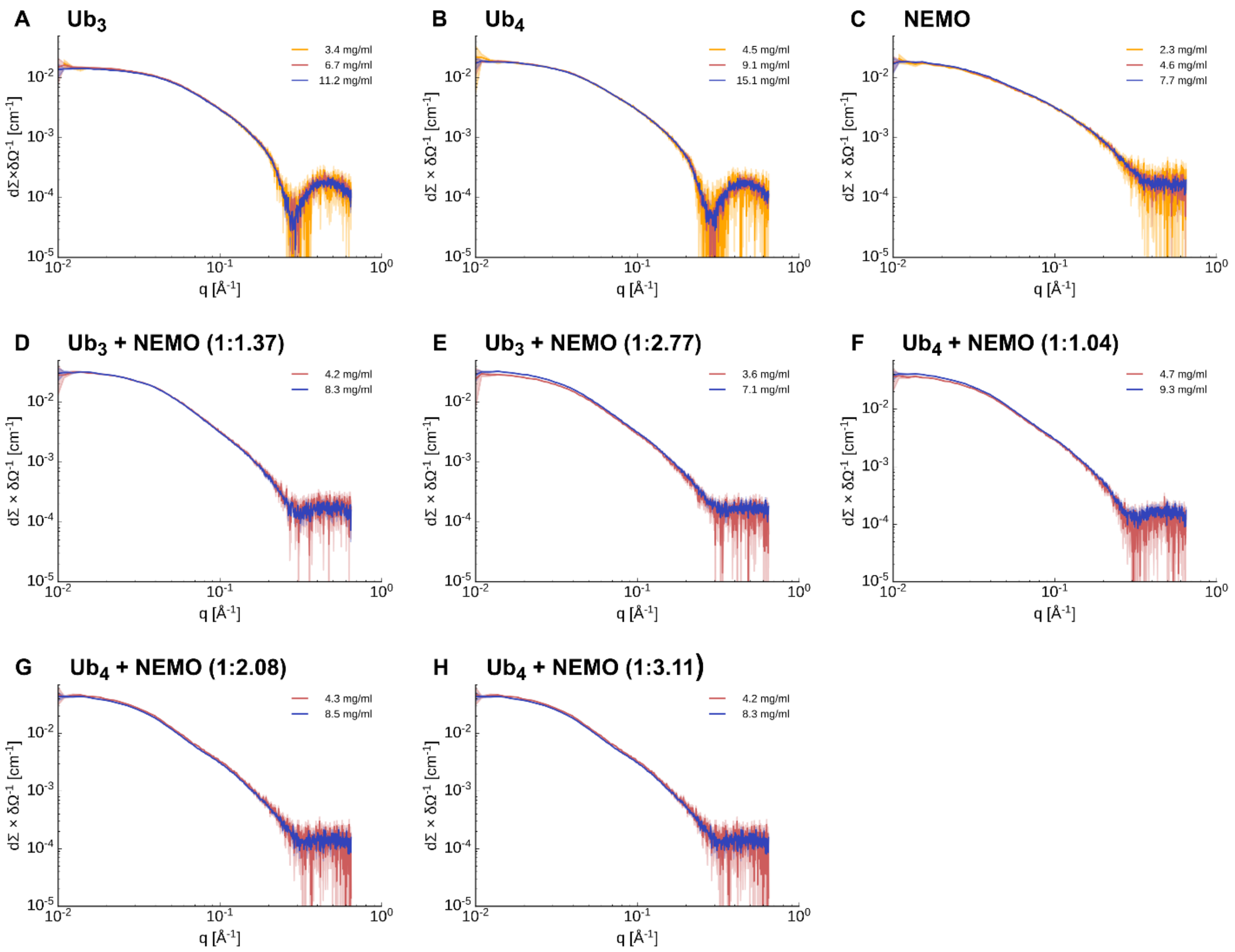

I

System	ratio	c (mg/ml)	corrected c*	NEMO / uM	Ub_x / uM
NEMO		7.72		702.5	
NEMO		4.62		420.4	
NEMO		2.34		212.9	
Ub_3		11.17			435.2
Ub_3		6.72			261.9
Ub_3		3.41			133.0
Ub_4		10.2	15.1		441
Ub_4		6.14	9.1		265
Ub_4		3.59	4.5		131

J

System	ratio	c (mg/ml)	corrected c*	NEMO / uM	Ub_x / uM
NEMO:Ub_3	1.37:1	8.29		279.4	203.5
NEMO:Ub_3	1.37:1	4.19		141.0	102.8
NEMO:Ub_3	2.77:1	7.12		351.2	126.9
NEMO:Ub_3	2.72:1	3.59		175.6	64.5
NEMO:Ub_4	1.04:1	7.01	9.25	210.2	202.9
NEMO:Ub_4	1.04:1	3.54	4.67	106.4	102.2
NEMO:Ub_4	2.08:1	6.83	8.48	308.7	148.6
NEMO:Ub_4	2.06:1	3.04	4.27	154.3	75.1
NEMO:Ub_4	3.11:1	6.97	8.30	377.8	121.4
NEMO:Ub_4	3.11:1	3.48	4.15	188.9	60.7

Fig. S5. Experimental SAXS curves with different protein concentrations. A) Linear Ub_3 **B)** linear Ub_4 **C)** NEMO **D)** Ub_3 with NEMO in a 1:1.37 ratio **E)** Ub_3 with NEMO in a 1:2.77 ratio **F)** Ub_4 with NEMO in a 1:1.04 ratio **G)** Ub_4 with NEMO in a 1:2.08 ratio **H)** Ub_4 with NEMO in a 1:3.11 ratio **I-J)** Concentrations of substrates used for SAXS measurements. Protein concentration (c) was determined by measuring the absorbance at 205 nm using specific absorbance for $NEMO_{258–350}$ C347S of 300990 M^{-1} cm^{-1}. *The concentration of Ub_4 was corrected based on SEC-SLS.

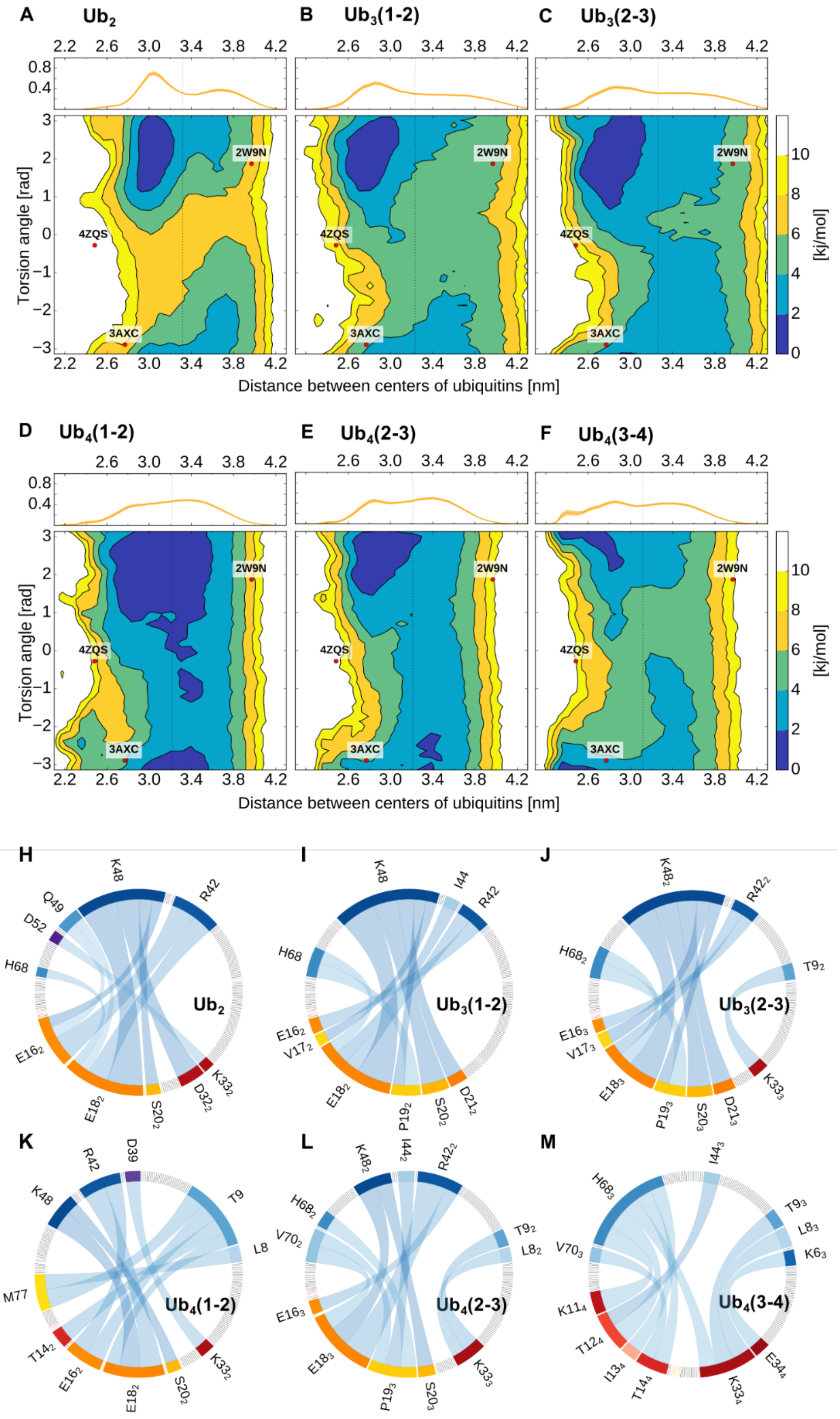

Fig. S6. Interactions between all neighboring ubiquitin pairs of Ub_2, Ub_3, and Ub_4. A-F) Free energy landscapes (in kJ/mol) as a function of the distance between the centers of the two ubiquitin domains and their relative orientation. The dots represent the coordinates associated with the available di-ubiquitin crystal structures. On top is shown the probability distribution of the distance between the centers of the two ubiquitin domains. **H-M)** Chord diagram of the

ten most common contacts between two close-by ubiquitins**.** The edge colors are corresponding to the colors of the interaction surfaces shown in **Figure 4d**. The thickness of the edge-edge connection is correlated to the probability of finding this specific interaction (thicker = more likely). The transparency of the connections depends on the type of interaction Low, intermediate or high transparency is related to charged, polar or hydrophobic interactions.

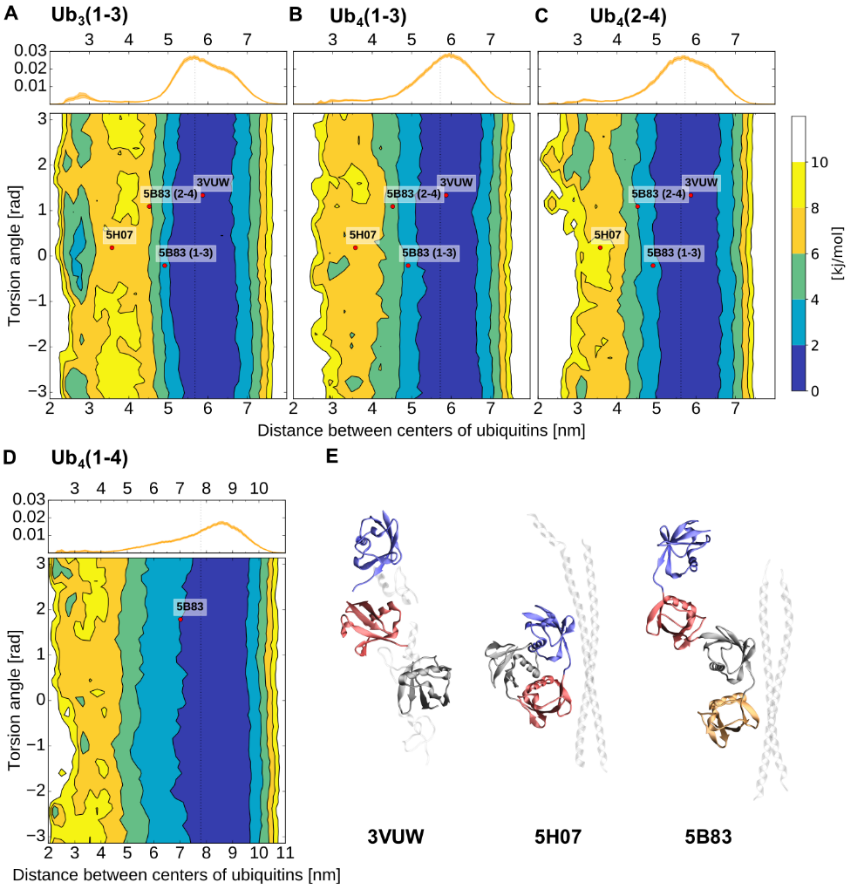

F

	Coulomb interaction [kJ/mol]	Lennard Jones interaction [kJ/mol]	Total [kJ/mol]	Ratio Coulomb interaction [%]
Di-ubiquitin				
Ub(1-2)	-4.3 ± 0.2	-11.7 ± 0.9	-16.0 ± 0.9	26.8 ± 2.0
Tri-ubiquitin				
Ub(1-2)	-6.8 ± 0.4	-37.8 ± 2.2	-44.6 ± 2.2	15.2 ± 1.1
Ub(2-3)	-5.7 ± 0.3	-36.1 ± 2.3	-41.8 ± 2.4	13.7 ± 1.0
Ub(1-3)	-0.8 ± 0.2	-6.6 ± 1.7	-7.5 ± 1.7	11.4 ± 3.7
Tetra-ubiquitin				
Ub(1-2)	-4.8 ± 0.3	-33.9 ± 2.5	-38.7 ± 2.5	12.5 ± 1.1
Ub(2-3)	-4.6 ± 0.3	-32.2 ± 2.2	-36.7 ± 2.2	12.4 ± 1.1
Ub(3-4)	-5.4 ± 0.3	-54.7 ± 4.4	-60.1 ± 4.4	9.0 ± 0.8
Ub(1-3)	-0.2 ± 0.1	-1.4 ± 0.6	-1.7 ± 0.6	14.3 ± 7.6
Ub(2-4)	-0.3 ± 0.1	-1.6 ± 0.8	-1.9 ± 0.8	13.9 ± 7.6
Ub(1-4)	-0.2 ± 0.1	-1.7 ± 1.0	-1.9 ± 1.0	9.8 ± 7.5

Fig. S7. Free energy surfaces for all non-neighboring ubiquitin pairs of Ub_3 and Ub_4. A-D) Free energy landscapes (in kJ/mol) as a function of the distance between the centers of the two ubiquitin domains and their relative orientation. The dots represent the coordinates associated with the available di-ubiquitin crystal structures. On top is shown the

probability distribution of the distance between the centers of the two ubiquitin domains. **E)** Crystal structures of linear Ub_3 and Ub_4 in a bound conformation (3VUW, 5H07, 5B83). **F)** Decomposition of the average interaction energy between ubiquitin pairs (residue 2-70).

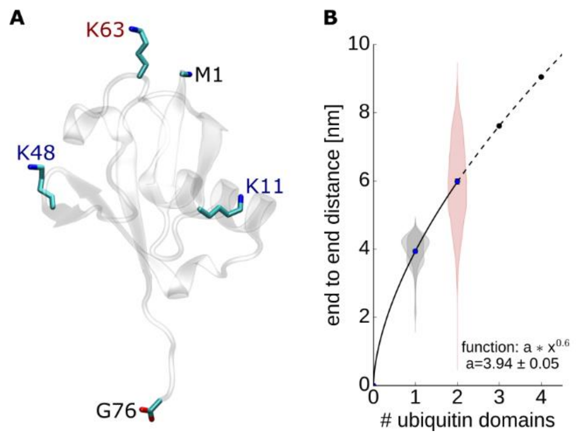

Fig. S8. End to end distances in ubiquitin. A) Start and end residues in the ubiquitin crystal structure. K63 side chain is further away from G76 than M1 backbone. K48 and K11 are closer to G76. **B)** Average end to end (e2e) distance of K63 poly-ubiquitin chain. The end to end (e2e) distance of N=1 was determined as the average e2e distance of the proximal and distal ubiquitin in K63 di-ubiquitin.

A
B

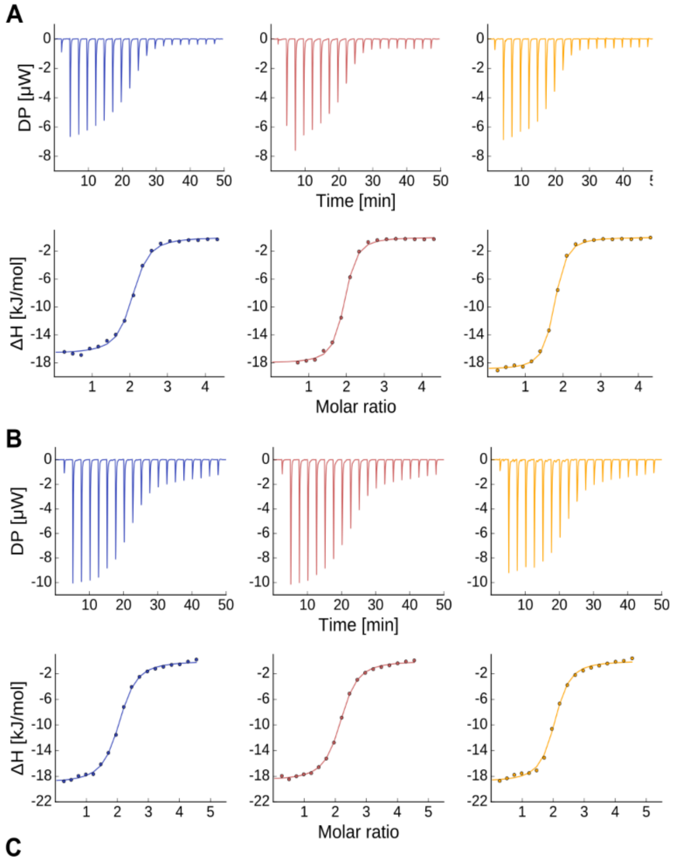

D
E

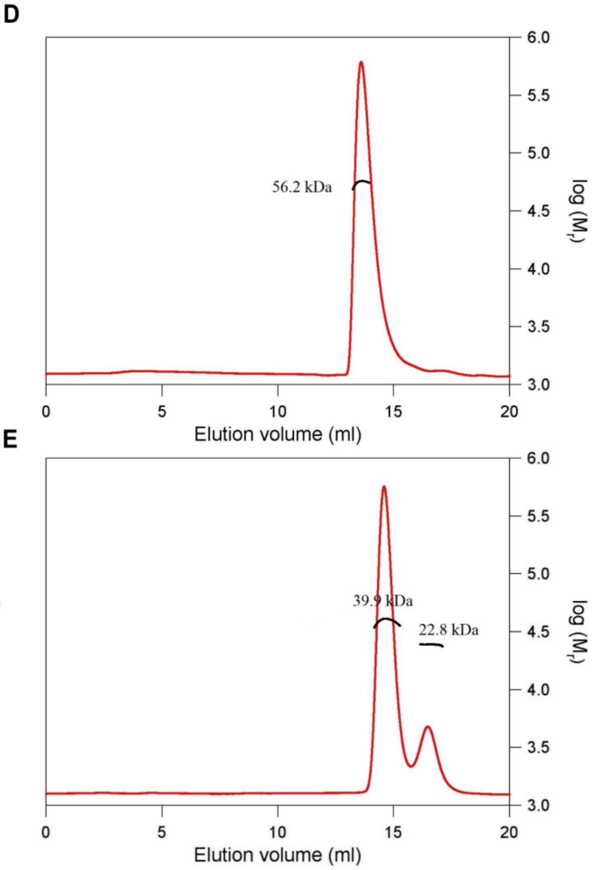

C

System	N	KD (µM)	ΔH (kJ/mol)	-TΔS (kJ/mol)
Ub3 + NEMO				
run 1	1.99 ± 0.01	2.14 ± 0.26	-16.7 ± 0.23	-15.7
run 2	1.85 ± 0.01	1.25 ± 0.14	-18.0 ± 0.21	-15.7
run 3	1.68 ± 0.01	1.33 ± 0.11	-18.9 ± 0.16	-14.6
**mean ± std error**	**1.84 ± 0.03**	**1.57 ± 0.28**	**-17.9 ± 0.64**	**-15.3 ± 0.4**
Ub4 + NEMO				
run 1	1.99 ± 0.01	4.66 ± 0.40	-19.0 ± 0.23	-11.4
run 2	2.10 ± 0.01	4.12 ± 0.29	-18.6 ± 0.17	-12.1
run 3	1.97 ± 0.02	3.61 ± 0.49	-18.8 ± 0.32	-12.2
**mean ± std error**	**2.02 ± 0.04**	**4.13 ± 0.30**	**-18.8 ± 0.1**	**-11.9 ± 0.3**

F
G

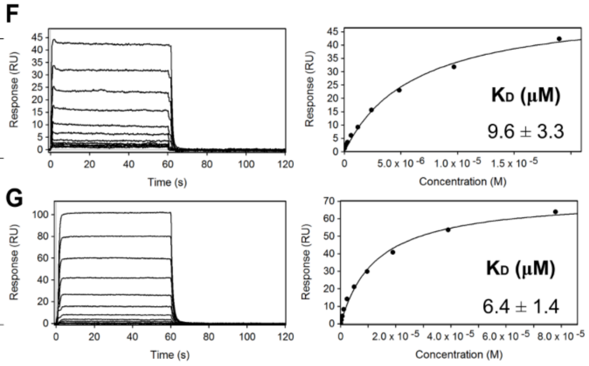

Fig. S9. ITC, SEC+SLS and SPR experiments. A-B) Isothermal titration calorimetry (ITC) measurement of the interaction of $NEMO_{258–350}$ with Ub_3 (A) and Ub_4 (B). **C)** Thermodynamic properties obtained by ITC with N for stoichiometry, K_D for dissociation constant, ΔH for enthalpy change and –TΔS for entropy change. **D-E)** Determination of the molecular weight of NEMO:Ub_3 (D) and NEMO:Ub_4 (E) complexes using size exclusion chromatography (SEC) in combination with static light scattering (SLS). The refractive index (red) and right-angle light scattering (not shown) signals were monitored and used to determine the molecular weights (black). The NEMO-Ub_3 sample elutes in two peaks. The first peak elutes at 14.6 ml and a molecular weight (MW) of 39.9 kDa containing a mixture of the NEMO:Ub_3 complex and free NEMO dimer. The second peak elutes at 16.5 ml and contained the remaining Ub_3 (MW of 24.6 kDa). The NEMO:Ub_4 complex eluted in one major peak at 13.6 ml and a MW of 56.2 kDa indicating that

indeed a 2:1 complex is formed. **F-G)** SPR sensorgrams of NEMO interacting with immobilized Ub_3 or Ub_4. NEMO was injected in two-fold serial dilution ranging from 0.9 –19 µM over immobilized Ub_3 (456 RU) (F) and Ub_4 (721 RU) (G) (Left panels). Sensorgrams are blank injection and reference surface subtracted. An activated and inactivated blank surface was used as a reference. Right panels display plots of equilibrium binding responses at the end of the analyte injections from sensorgrams in left panels against analyte concentration. Steady state equilibrium dissociation constants (K_D) is fitted from curves and a 1:1 model.

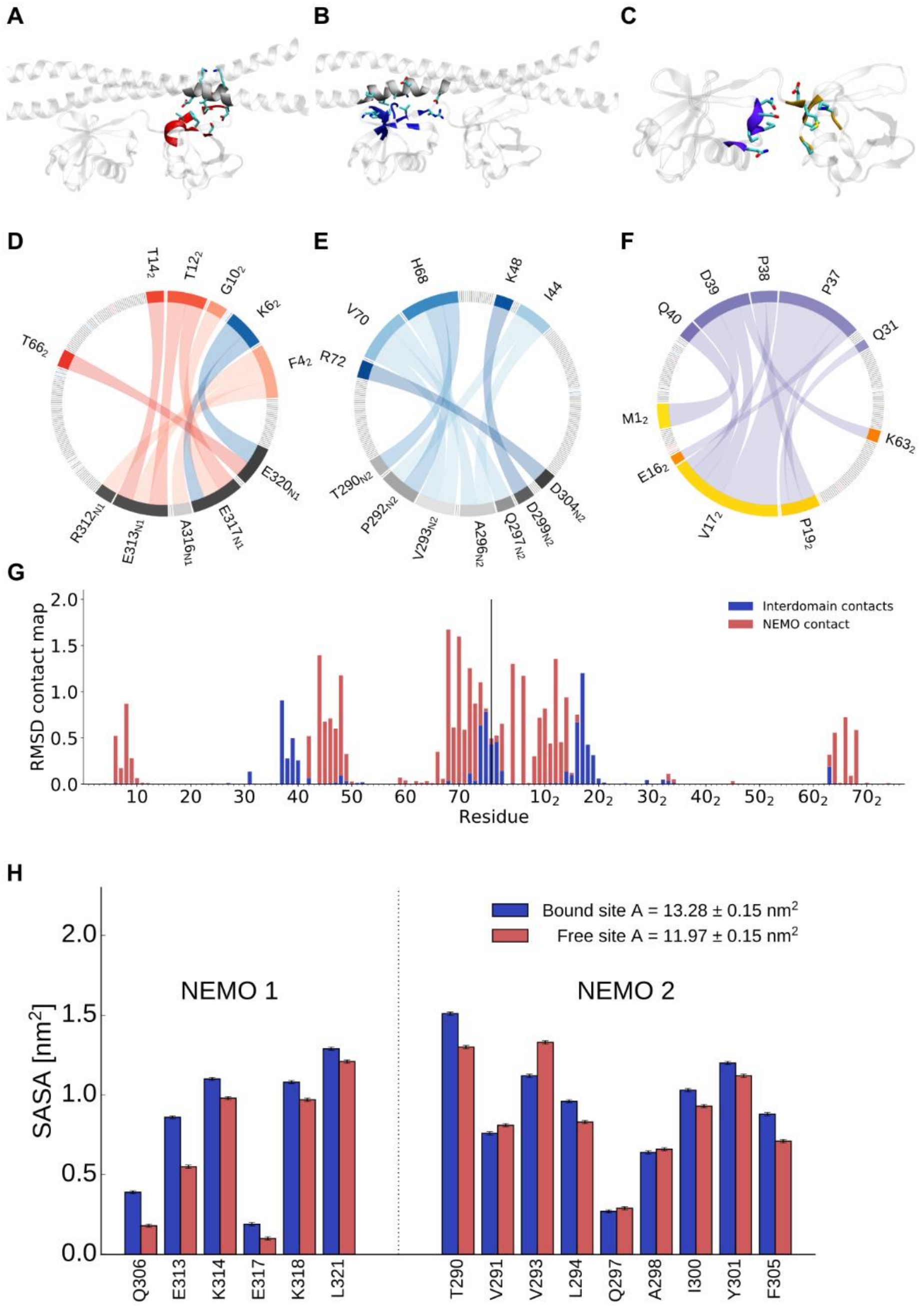

Fig. S10. Intramolecular interactions of di-ubiquitin and NEMO. A-B) Interaction surfaces of Ub_2 and NEMO 1 (A) and of Ub_2 and NEMO 2 (B). The red and blue surface is similar to the blue area marked in **Figure 4d**. **C)** Interaction surface of two neighboring ubiquitins. **D-F)** Chord diagram of the ten most common contacts between NEMO 1 (D) and Ub_2, NEMO 2 and Ub_2 (E), and both ubiquitin cores (F). The edge colors are corresponding to the colors of the interaction surfaces shown in A-C. **G)** Contact map differences between free and NEMO-bound Ub_2. **H)** Solvent accessible surface area (SASA) changes in NEMO dimer upon binding to Ub_2. The bars show the SASA per residue, averaged over the conformational ensemble, for the residues involved in the binding with Ub_2. Blue bars are for the two NEMO monomers in the ubiquitin bound state, while the red bars are for the free state. On top is reported the SASA for all the considered residues.

Table S1. Overview of all simulations.

System	Total number of beads	Metadynamics	Metainference (SAXS)	Number of beads for SAXS calculation	Number of replicas	Total Simulation length [μs] (simulation length per replica [ns])
Di-ubiquitin						
**Martini 2.2**	13569	x			112	48 (428)
**Increased P-W interaction**	13569	x			112	69 (616)
**With SAXS**	13569	x	x	355	112	65 (581)
**With SAXS(every time step)**	13569	*x*	x	355	112	58 (517)
**Martini 3 beta**	13569				112	242 (2160)
Tri-ubiquitin						
**Increased P-W interaction**	12501	x			112	99 (884)
**With SAXS**	12501	x	x	489	112	60 (536)
Tetra-ubiquitin						
**Increased P-W interaction**	15445	x		652	112	60 (536)
**With SAXS**	15445	x	x	652	112	63 (562)
Nemo						
**With SAXS**	33698	*x*	x	400	64	8 (125)
K63 Ub_2						
**Increased P-W interaction**	13151	*x*			112	72 (641)
**With SAXS**	13151	*x*	*x*	328	112	62 (554)
**With SAXS and weaker elastic network**	13151	*x*	*x*	328	112	73 (651)
**With SAXS and +10% increased P-W interaction**	13151	*x*	*x*	328	112	64 (572)
**Excluded volume**	328				112	19 (173)
**Excluded volume + SAXS**	328		*x*	328	112	11 (102)

Table S2. Determination of the molecular weight of NEMO, Ub_3, Ub_4, NEMO-Ub_3 and NEMO-Ub_4 using size exclusion chromatography (SEC) in combination with static light scattering (SLS). Apart from indicated with "#" the conditions were 50 mM Tris.HCl pH 8, 300 mM NaCl. # 50 mM sodium phosphate pH 7, 50 mM NaCl (ITC conditions). * Peaks are not fully separated. Peak of complex "integrated".

Protein	Peak 1 / ml (MWexp / kDa)	Peak 2 / mL (MWexp / kDa)	MWcalc /kDa	Remarks
**NEMO dimer**	15.5 (**20.6**)	-	**22.0**	-
**Ub_3**	16.6 (**24.1**)	-	**25.7**	-
**Ub_4**	15.9 (**32.3**)	-	**34.2**	-
**NEMO:Ub_3 1.4:1**	14.6 (**39.9**) **(45.0)***	16.5 (**24.6**)	**47.7 (2:1)**	Excess Ub_3
**NEMO:Ub_3 2:1**	14.7 (**41.1**)	17.0 (**22.8**)	**47.7 (2:1)**	Free Ub_3
**NEMO:Ub_3 2:1#**	14.2 (**42.2**)	-	**47.7 (2:1)**	-
**NEMO:Ub_3 4:1**	14.6 (**40.8**)	15.3 (**24.1**)	**69.6 (4:1)**	Excess NEMO
**NEMO:Ub_4 1.0:1**	13.7 (**53.0**)	15.8 (**32.7**)	**56.2 (2:1)**	Excess Ub_4
**NEMO:Ub_4 2.0:1**	13.6 (**56.2**)	-	**56.2 (2:1)**	No extra peaks.
**NEMO:Ub_4 4.1:1**	13.6 (**53.2**)	15.2 (**21.3**)	**78.2 (4:1)**	Excess NEMO
**NEMO:Ub_4 2.5:1#**	13.1 (**53.4**)	14.6 (**16.5**)	**78.2 (4:1)**	Excess NEMO
